# Changes in Endogenous Essential Metal Homeostasis in the Liver and Kidneys during a Six-Month Follow-Up Period after Subchronic Cadmium Exposure

**DOI:** 10.3390/ijms25073829

**Published:** 2024-03-29

**Authors:** Rafał Kusak, Marzenna Nasiadek, Joanna Stragierowicz, Wojciech Hanke, Anna Kilanowicz

**Affiliations:** 1 Medical Centers the Medici, 91-053 Lodz, Poland; 2 Department of Toxicology, Medical University of Lodz, 90-151 Lodz, Poland; joanna.stragierowicz@umed.lodz.pl (J.S.); anna.kilanowicz@umed.lodz.pl (A.K.); 3 Medical Informatics and Statistics Department, Medical University of Lodz, 90-419 Lodz, Poland; wojciech.hanke@umed.lodz.pl

**Keywords:** cadmium, essential elements, kidney, liver, plasma, rats, toxicity

## Abstract

Cadmium (Cd) is one of the most dangerous environmental pollutants. Its mechanism of action is multidirectional; among other things, it disrupts the balance of key essential elements. The aim of this study was to assess how cumulative exposure to Cd influences its interaction with selected essential elements (Cu, Zn, Ca, and Mg) in the kidney and liver during long-term observation (90 and 180 days) after subchronic exposure of rats (90 days) to Cd at common environmental (0.09 and 0.9 mg Cd/kg b.w.) and higher (1.8 and 4.5 mg Cd/kg b.w.) doses. Cd and essential elements were analyzed using the F-AAS and GF-AAS techniques. It was shown that the highest bioaccumulation of Cd in the kidney occurred six months after the end of exposure, and importantly, the highest accumulation was found after the lowest Cd dose (i.e., environmental exposure). Organ bioaccumulation of Cd (>21 μgCd/g w.w. in the kidney and >6 μgCd/g w.w. in the liver) was accompanied by changes in the other studied essential elements, particularly Cu in both the kidney and liver and Zn in the liver; these persisted for as long as six months after the end of the exposure. The results suggest that the critical concentration in human kidneys (40 μgCd/g w.w.), currently considered safe, may be too high and should be reviewed, as the observed long-term imbalance of Cu/Zn in the kidneys may lead to renal dysfunction.

## 1. Introduction

Environmental pollution with heavy metals and its associated exposure is a growing global problem worldwide. Among these, cadmium (Cd) is particularly widespread, mainly due to anthropogenic activities, such as the production of metals, pesticides, and phosphate fertilizers, and fossil-fuel combustion, as well as its use in Cd–Ni batteries, photovoltaic modules, paints, plastics, and electroplating [1]. Due to its persistent character and high bioaccumulation rate in living organisms, as well as its toxicity, it has been ranked seventh among environmentally hazardous substances and toxic substances with a high risk of disease [2].

The main sources of exposure of the general population to Cd and its compounds are the consumption of contaminated food by the non-smoking population and smoking among the smoking population [1,3]. Its absorption ranges from 5 to 15% by the oral route and from 30 to 50% by the inhalation route (for smokers) [1]. A large body of evidence suggests that the lower iron levels in women, especially those of childbearing age, contribute to higher absorption of Cd; hence, this group is at particular risk of exposure [4,5]. After Cd is absorbed into the body, it is transported in the bloodstream by erythrocytes and albumin; it eventually accumulates mainly in the kidneys and liver, with the concentration increasing with age [6,7]. Cadmium is characterized by a long biological half-life, i.e., 10–38 years, depending on the tissue, and its elimination from the body is a slow process [6,8]. These properties will exacerbate the toxic effects of Cd, especially in the target organs (kidneys and liver), although studies in recent years have also demonstrated that Cd has harmful effects on bones, the reproductive organs, the circulatory system, and the nervous system [4,7,8,9,10].

In the case of chronic exposure to repeated high or moderate Cd levels, the target site is the kidneys, which can even lead to generalized proximal tubule dysfunction [1,4,7,11,12]; however, most of the general population in industrialized countries is exposed regularly to low levels of Cd, and it is unclear how this could affect health [7]. The second organ is the liver, where the metal induces the synthesis of low-molecular-weight proteins, metallothioneins (MTs), that buffer the toxic effects of Cd by binding to it [8,13]. While it was previously thought that environmental exposure to Cd primarily damaged the kidneys, a growing body of research now shows that it may also be a contributing factor to liver dysfunction [14,15]. Lifetime exposure to Cd, even at the low doses found in the environment, contributes to the burden of this metal throughout the body. In addition, Cd is classified by the IARC (International Agency for Research on Cancer) as a carcinogen in humans (Group I) [16].

Many in vitro and in vivo studies indicate that the mechanism of Cd toxicity is multidirectional and closely related to its affinity for protein structures containing –SH groups, i.e., proteins, enzymes, and nucleic acids. Indeed, their structure and functions have been found to be altered by Cd [8,17]. Exposure to Cd can also increase oxidative stress via the induction of reactive oxygen species (ROS) or reduction of cellular antioxidant defenses: catalase, manganese superoxide dismutase, and cooper-zinc superoxide dismutase [18,19]. It is possible that oxidative stress caused by Cd may be one of the key mechanisms responsible for liver and kidney disease. It is known that essential elements such as zinc (Zn), copper (Cu), calcium (Ca), and magnesium (Mg) are closely related to oxidation processes and can directly or indirectly protect cells from oxidative damage [20]. Many studies indicate the presence of interactions between Cd and key essential elements (Cu, Zn, Ca, and Mg) [21,22,23,24,25,26,27], although it is unknown whether the resulting changes are long-lasting. 

Hence, this study attempts to determine the length of time that Cd continues to disrupt essential element homeostasis in a dose-dependent manner after the end of 90 days of exposure. This study employs the same exposure model as used previously [28,29]; those studies proved that Cd continued to induce persistent oxidative stress and disorders in the female reproductive system for up to six months after exposure: these manifest as endometrial thickness changes, hormonal changes, and estrous cycle disorders [29]. However, no data currently exist on the levels of Cd and essential elements in the liver and kidney after a long period following the termination of Cd exposure. 

To obtain these data, this study uses extended observation periods (three and six months) following subchronic exposure to Cd at doses similar to environmental exposure and higher. Exposure to rats for several weeks (90 days in our study) is assumed to be equivalent to several months of exposure in humans [30]. The daily doses of Cd used in this study correspond to environmental doses (0.09 and 0.9 mg/kg b.w.) and higher (1.8 and 4.5 mg/kg b.w.), and the per os route used reflects exposure from food, the main source of Cd for the non-smoking general population. 

The aim of this study was to determine the interaction of Cd with selected essential elements (Cu, Zn, Ca, and Mg) in the kidney and liver during long-term follow-up (three and six months) after subchronic exposure (90 days) to Cd at environmental (0.09 and 0.9 mg Cd/kg b.w.) and higher (1.8 and 4.5 mg Cd/kg b.w.) doses.

## 2. Results

### 2.1. Clinical Signs, Food and Water Consumption

No macroscopic or behavioral abnormalities or any unexpected deaths were noted in any of the rat groups throughout the experiment. No significant differences in feed or water consumption were found between any of the study groups and their respective control groups (Supplementary Tables S1–S6).

### 2.2. Body Mass and Organs Weight

The measurements of initial and terminal body weight, as well as relative and absolute weights of kidneys and liver, are presented in Supplementary Table S7. The obtained results did not show any statistically significant differences between the study groups and their respective control groups.

### 2.3. Cd in Plasma, Kidneys, and Liver

**Figure 1:**
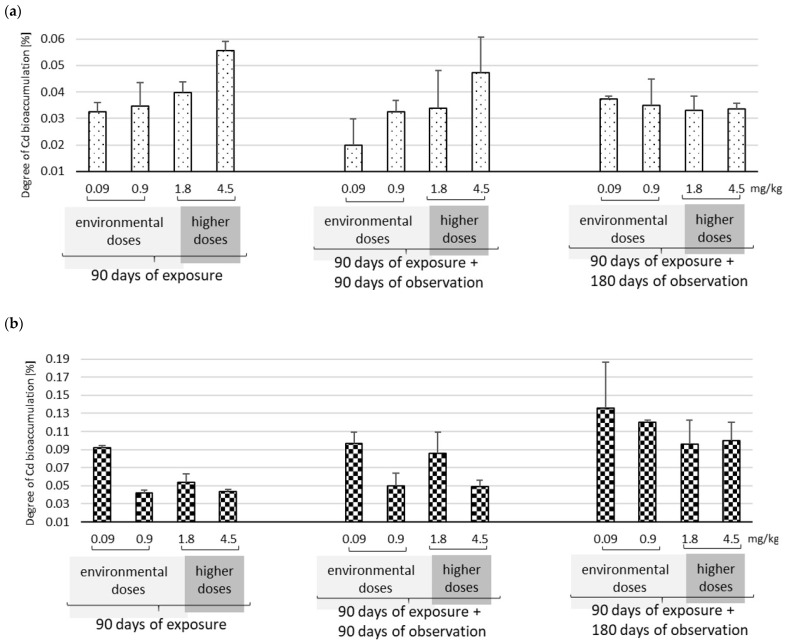
Cd bioaccumulation in rat liver (**a**) and kidneys (**b**) at the end of 90 days per os Cd administration and at the two post-exposure observation points (90 and 180 days). Shaded curves show higher Cd doses (1.8 and 4.5 mg/kg b.w.); unshaded show environmental Cd doses (0.09 and 0.9 mg/kg b.w.).

The Cd concentrations in plasma, liver, and kidneys after 90 days of oral exposure to Cd and after a 90 days or 180 days of observation period are presented in Table 1 and Figure 1. The 90 days of exposure to Cd at four different doses (0.09–4.5 mg/kg b.w.) was associated with higher and dose-dependent Cd concentrations in all analyzed samples of plasma, liver, and kidneys. The ratio between Cd in plasma and Cd in whole blood was calculated based on the Cd levels previously obtained in whole blood [31]. The result indicates that directly after exposure, the amount of Cd in the plasma increases proportionally with Cd exposure. Higher Cd concentrations were noted in the liver and kidneys than in the plasma. The obtained levels were also dependent on the Cd dose (Table 1).

Ninety days after the end of exposure, changes in Cd levels were observed in the examined tissues. The most significant decrease in Cd levels was recorded in plasma. However, the ratio of Cd concentration in plasma to whole blood was approximately 50%, irrespective of the dose over time. In the liver, only the highest dose (4.5 mg/kg b.w.) yielded a significant reduction in Cd levels after 90 days compared to immediately after exposure; the three smaller doses (0.09–1.8 mg/kg b.w.) resulted in no change in Cd levels over the observation period. Cd levels in the kidneys were characterized by a greater or lesser increase after the same period. No reduction in renal Cd concentration was observed at any dose.

Following a further 90 days of observation (i.e., 180 days in total), a further decrease in plasma Cd levels was noted compared to the previous observation period. This decrease was particularly marked for the two lowest doses (0.09–0.9 mg/kg b.w.), i.e., comparable to environmental doses. Higher Cd doses (1.8–4.5 mg/kg b.w.) resulted in the Cd concentration in plasma being maintained regardless of the observation period (90 or 180 days). However, at the higher doses, the Cd plasma:whole blood ratio increased significantly, i.e., the opposite of the results obtained immediately after the end of exposure. In the liver, the Cd levels remained at a similar level (for the three lower doses) or slightly decreased (at the highest dose: 4.5 mg/kg b.w.) after 90 and 180 days of observation compared to the end of exposure. In the kidneys, all tested Cd levels were still found to increase compared to the end of the exposure period and the first 90 days post-exposure observation point.

In the liver, Cd accumulation increases and decreases regardless of dose and observation time. However, after the longest observation period (180 days), all used doses of Cd demonstrate very similar accumulation, i.e., approximately 3.5 × 10^–4^ μg/g tissue (0.03%). 

### 2.4. Zn, Cu, Ca, and Mg Concentrations

**Figure 2:**
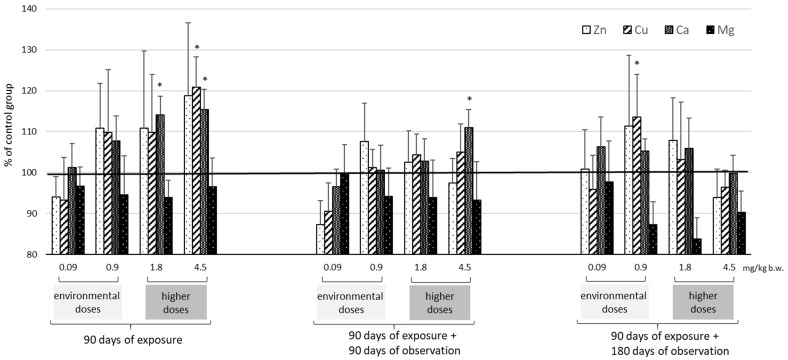
Changes in the levels of selected trace elements (Zn, Cu, Ca, and Mg) in rat plasma given in relation to mean concentration of controls (marked line 100%) after 90 days of oral exposure to Cd and after 90 and 180 days post-exposure; *p* ≤ 0.05 (* vs. control group).

**Figure 3:**
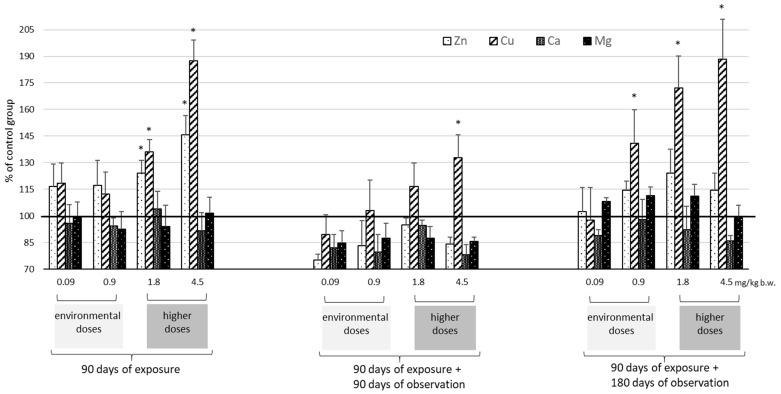
Changes in the levels of selected trace elements (Zn, Cu, Ca, and Mg) in rat kidney in relation to mean concentration of controls (marked line 100%) after 90 days of oral exposure to Cd and after 90 and 180 days post-exposure; *p* ≤ 0.05 (* vs. control group).

**Figure 4:**
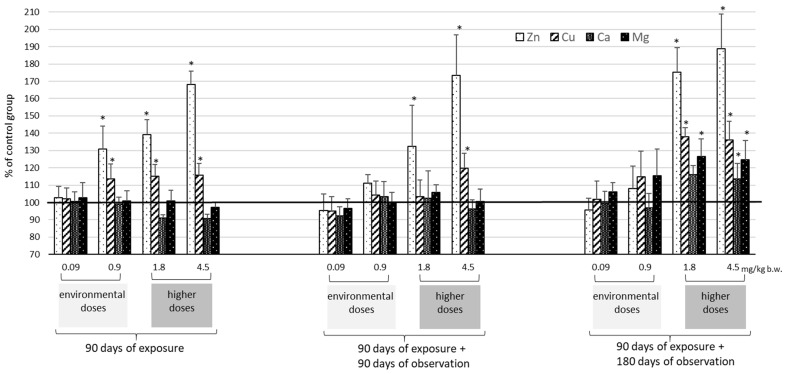
Changes in the levels of selected trace elements (Zn, Cu, Ca, and Mg) in rat liver in relation to mean concentration of controls (marked line 100%) after 90 days of oral exposure to Cd and after 90 and 180 days post-exposure; *p* ≤ 0.05 (* vs. control group).

The identified levels of selected essential elements (Zn, Cu, Ca, and Mg) in the plasma, kidneys, and liver are presented as absolute values in Table 2 and Table 3 and as % of control values in Figure 2, Figure 3 and Figure 4. These findings indicate that exposure to Cd has the greatest effect on Zn and Cu levels and less so on Ca and Mg.

In the plasma, kidney, and liver, Zn and Cu levels typically increased after exposure to Cd (Table 2, Figure 2, Figure 3 and Figure 4). The most marked increases in Zn and Cu levels were observed in the liver and kidneys, particularly at the higher Cd doses (1.8–4.5 mg/kg b.w.). Also importantly, after 180 days of observation, kidney Cu concentrations increased significantly (0.9 mg/kg b.w.) with higher Cd concentration (>23 μg/g wet weight). In the kidneys, the Cu/Zn ratio increased with higher Cd dose and observation period length (90 and 180 days); in the liver, the ratio increased with exposure time but decreased with increasing Cd dose.

For Ca and Mg, only a few statistically significant changes were recorded in the analyzed tissues (Table 3, Figure 2, Figure 3 and Figure 4). The most marked increase in both Ca and Mg was visible only after 180 days of observation after exposure to the highest two doses of Cd (1.8–4.5 mg/kg b.w.).

## 3. Discussion

The past century has seen a manifold increase in renal Cd concentrations in the general population, with the most important health effect being renal tubular damage [8,9,12]. There are also numerous reports that environmental exposure to Cd can also lead to hepatotoxicity [14,15]. Findings in both animals and humans indicate that chronic exposure to Cd induces multimodal toxicity in various organs, with one route involving interactions with essential elements (Cu, Zn, Ca, Mg, and Fe), particularly Zn and Cu [21,26,27]. However, it has not been verified whether such disruption of essential element homeostasis may persist for a long period after the end of subchronic exposure to Cd. 

Therefore, this study examined the effect of exposure to Cd at environmental doses (0.09 and 0.9 mg/kg b.w.) and at two higher levels (1.8 and 4.5 mg/kg b.w.), which did not disturb the integral toxicity indices. The aim of this study was to assess both the organ distribution (liver and kidneys) of Cd and the concentration of trace elements in plasma, kidneys, and the liver in a long-term observation model lasting three and six months after the end of a three-month exposure period. 

Our findings indicate that immediately after exposure, Cd accumulates mainly in the liver or kidneys in a dose-dependent manner; this is consistent with the results of other authors [25,27,32,33]. In addition, thanks to our research model, our data indicate dose-dependent organ redistribution, both immediately after the end of exposure and during follow-up: kidney Cd concentration was up to three times higher than in the liver at the environmental doses, while higher concentrations were noted in the liver at the highest dose (4.5 mg/kg b.w.). Similarly, Brzóska et al. (2015) also reported higher accumulation in the kidneys than the liver at approximately environmental doses; however, unlike our study, they did not evaluate these levels during an observation period following the termination of Cd administration [25]. 

Similarly, previous research found that a single high dose of Cd via the oral route resulted in higher accumulation in the liver than the kidneys immediately after exposure. However, an observation period lasting several months found the Cd to be redistributed with higher concentrations in the kidney than in the liver [6]. Also, in this study, a significant (two- to three-fold) increase in renal Cd concentration was observed six months after the end of subchronic exposure, regardless of the dose. However, the highest accumulation of Cd was noted in the kidneys, i.e., a two- to three-fold increase at the lowest environmental dose; no similar effect was noted for the liver. These observations are in line with post-mortem studies of Cd concentrations in the general population, which showed nearly 10 times higher concentrations in the kidneys than in the liver [34,35,36].

The increase in renal Cd concentrations noted during the observation period is due to the redistribution of Cd from the liver to the kidneys. Previous studies have shown that Cd, after absorption, is initially transported to the liver, where it induces the synthesis of Cd-binding MT. Later, as the hepatocytes die, Cd is released from the Cd–MT complex and enters the kidneys with the bloodstream [13,37]. Our study also found that the redistribution of Cd from the liver to the kidneys depends on the amount of exposure. At high exposures, up to 40% reductions in liver Cd concentrations were noted with observation time, up to six months, while after lower environmental doses, liver Cd concentrations increased by about 20%. Hence, it can be cautiously concluded that at low doses, the significant increase in renal Cd concentration noted during the observation period is mainly due to its redistribution from the plasma, where it is bound to proteins or other molecules with SH groups [38]. Many studies indicate that Cd can enter the kidneys both directly from the plasma by uptake of Cd–SH conjugates, as well as through channels and transporters of ions such as Ca^2+^ or Zn^2+^ [38,39]. 

Our findings show a significant reduction in plasma Cd concentrations by up to 95% after the longest observation period at environmental doses. As these low doses also demonstrate the highest degree of accumulation in the kidneys, it can be concluded that even low-level Cd exposure may pose a significant risk of kidney damage in the general population. Although our study was performed on rat kidneys, our findings remain the only such data acquired in vivo, considering low levels of lifetime exposure comparable to the current exposure of the general population in industrialized countries. 

Cd redistribution can disrupt the homeostasis of essential elements in both the liver and kidneys and the body as a whole. Although the interaction of Cd with trace elements is well documented [21,22,23,24,25,26,40], the direction of these changes is not clear. This study showed that Cd accumulation after environmental exposure can disrupt the homeostasis of essential elements and can increase Zn and Cu levels in the liver. While these results are consistent with previous studies [21,22,23], others have also noted that exposure to low doses of Cd can lead to decreased Zn and Cu concentrations in the kidney and liver [26,41,42]. 

After higher doses, increased Zn and Cu concentrations were observed in both the liver and kidneys, as noted previously [40,43,44]. Abnormalities in Zn homeostasis were noted mainly in the liver and Cu in the kidney; this is due to the fact that Cu-bound MT predominates in the kidney and Zn-bound MT predominates in the liver [13,45]. Importantly, our findings demonstrate for the first time that in both organs, the disturbances of Zn and Cu induced by Cd were long-lasting and persisted for at least six months post-exposure, depending on the dose administered. After exposure to environmental doses of Cd, its increasing accumulation in the kidneys over time after exposure resulted in an increase in Cu concentrations of about 40%, but only after six months. In contrast, administration of higher doses of Cd led to an increase in renal Cu levels immediately after exposure, which persisted throughout the observation period. In the liver, while disturbances in Cu and Zn homeostasis (increases) were already evident after exposure at all doses (0.9–4.5 mg/kg b.w.), these changes persisted throughout the observation period by as much as 50% (Cu) and 40% (Zn), but only for higher doses (1.8–4.5 mg/kg b.w.). Similar perturbations in Zn and Cu concentrations in relation to Cd concentration, viz. from 1 μgCd/g w.w. in the liver and from 26 μgCd/g w.w. in the kidney, were described in human tissues by Satarug et al. (2001) [46]. 

It is possible that the persistent increase in renal Cu concentrations, and thus disturbances in the Zn/Cu ratio, may be associated with renal dysfunction. Indeed, Ikeda et al. (2007) reported a stronger correlation between the β2-microglobulin concentration (an early marker of renal function) and the Cu concentration than with the Cd concentration in the urine of women environmentally exposed to Cd [47]. Also, Satarug et al. (2018) suggested that an imbalance between Cu and Zn is an independent risk factor for renal tubular damage through oxidative stress [7]. Cd induces the indirect progression of oxidative stress by undermining antioxidative protection mechanisms, thereby altering the electron transport chain in mitochondria, and by segregating certain ions, such as Cu and Fe, from proteins, which can directly stimulate ROS production (free radicals via the Fenton reaction) [48,49,50]. 

A number of experimental studies suggest that ROS-induced oxidative damage plays an important role in Cu toxicity, with in vivo evidence indicating that excess Cu promotes oxidative modification of low-density lipoprotein (LDL), atherogenesis [51], reduced catalase and GSH peroxidase activity, and increased mitochondrial lipid peroxidation products [52,53,54,55]. Hence, the presence of persistently elevated Cu concentrations in the kidneys, especially after low Cd exposure, may negatively impact these organs, as indicated previously [56]. This suggests that kidney damage may occur at lower Cd concentrations, but it is difficult to assess the Cd threshold level in the kidneys due to a lack of epidemiological data on renal concentrations in humans as assessed intravitally. It is assumed that the critical concentration of Cd in the renal cortex is ≥50 μg/g w.w., which currently threatens 10% of the population [8,57]. In these studies, the effects of homeostasis disturbances were observed in the rat kidneys at the concentration of 21 μg/g w.w., corresponding to the concentration of approximately 29 μg/g w.w. in the renal cortex (using the conversion factor 1.25). 

In contrast, few studies have examined whether the increase in Zn concentration in the liver induced by Cd may contribute to its hepatotoxicity. Some studies indicate that Zn acts as a protective factor against the action of ROS [19]; on the other hand, it is not impossible that a disturbed Cu/Zn balance may have a negative effect on hepatocytes. Gattea Al-Rikabi et al. (2021) reported that exposure to Zn at environmental doses in a rat model resulted in histopathological alterations in the liver, manifesting as a marked disarrangement of hepatic cords with compressed sinusoids, severe cellar swelling of hepatocytes, marked nuclear hypertrophy, and necrosis of hepatocytes with tissue depletion, as well as in the kidneys, characterized by mild vascular degeneration and necrosis of renal tubules with tubular cast formation [58]. In our study, the presence of persistently elevated Zn concentration in the liver and the related disturbed Cu/Zn balance may suggest hepatotoxicity.

Cd exposure also resulted in an increase in hepatic Ca and Mg concentrations six months after the end of exposure, but only at the two higher doses. This delayed effect of Cd is likely due to its accumulation in hepatocytes. Given the very similar physicochemical properties of Cd and Ca, Cd-induced cellular damage may be due to the displacement of Ca from various cellular proteins and signaling pathways [59], as well as the modulation of extracellular G protein-coupled receptors (CaSR), ryanodine (RyR) and inositol triphosphate receptors (ITPR) [60,61]. Persistently elevated Ca levels activate calmodulin (CAM) and calmodulin-dependent kinase II (CAMKII) proteins, which cause mitochondrial dysfunction and oxidative stress and, in turn, release mitochondrial cytochrome C (Cyt_C), initiating caspase (Casp)-dependent cell death [61,62]. These interactions with Ca play a key role in Cd toxicity, which most likely occurs through changes in CAM and CAMKII activation. An increase in serum Ca concentration was also observed after exposure, but only after higher doses of Cd were administered. The results obtained were consistent with the work of other authors, who additionally showed an increase in urinary Ca excretion [63]. However, women exposed to high doses of Cd (as in Itai-Itai), as well as those exposed to environmental levels of Cd, often showed a decrease in serum calcium concentration, which was not observed in animal models. It is important to remember that changes in women’s serum Ca concentration are influenced by a number of factors, including age, number of births, hormone balance (parathormone), vitamin D, and diet. Therefore, it seemed reasonable to use a long observation period not previously studied elsewhere to reveal potential long-term changes in levels of this element in animals. The lack of change, or even the increase in serum calcium levels observed by us in the experimental animals during the observation period after exposure to Cd, can probably be related to the direct effect of Cd on bone, which leads to reduced Ca incorporation into bone [63].

Interactions between Cd and Mg are less marked than their interactions with Zn, Cu, or Ca. Our present findings and those of previous studies confirm that Cd can interfere with Mg absorption in the gastrointestinal tract and affect its homeostasis in tissues [19]. Six months after the end of exposure, significant increases in Mg and Ca concentrations were observed in hepatocytes. Although such increases in Mg concentration have been noted by other authors [19,21,40], only immediately after the end of subchronic exposure, it is still difficult to predict the consequences associated with its excess in hepatocytes due to its range of important physiological and biochemical functions. 

Cd, besides its documented interactions with the bioelements studied in this work, can also interact with another important element, iron, which was not investigated in this study. A significant discovery was made by Tokumoto et al. [64], showing that long-term exposure to Cd (over 12 months) in mice leads to a decrease in Fe concentration in the liver and inhibits the expression of *HCP1* and *Cybrd1*. This indicates that iron absorption could be inhibited or significantly reduced despite its normal serum concentration. They suggest that prolonged exposure to Cd may exacerbate iron deficiency, leading to anemia, and that *HCP1* and *Cybrd1* may be key factors in Cd-induced iron deficiency.

## 4. Materials and Methods

### 4.1. Experimental Animals and Groups

This study used 120 female Wistar rats (aged 9–10 weeks old) obtained from the Laboratory Animal Center of the Medical University of Lodz. The animals were maintained in polypropylene steel cages under controlled conditions (12 h light/dark cycles at room temperature 22 ± 1 °C and relative humidity 50–60%) for two weeks before the beginning of the experiment. The rats had free access to water and a standard diet. 

**Figure 5:**
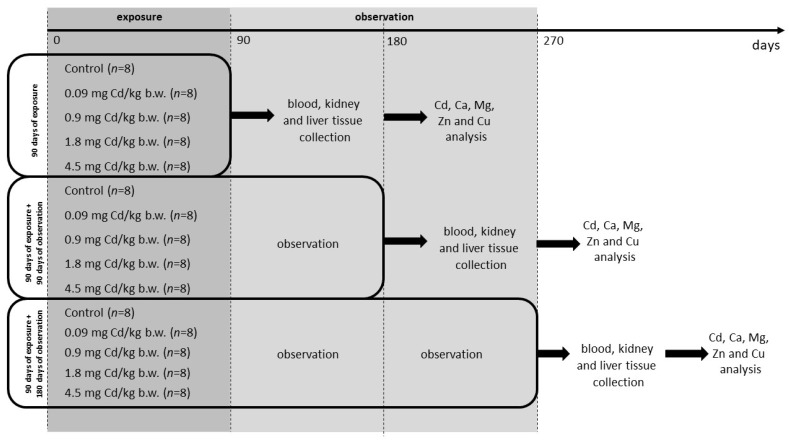
Plan of the experiment.

The experiments were conducted on female rats, which were divided randomly into three groups: the first receiving a 90 days of exposure to Cd, the second undergoing 90 days of observation after the 90 days of exposure, and the third undergoing 180 days of observation after the 90 days of exposure. All groups included a control group (*n* = 8) and a Cd-exposed group (*n* = 32). The Cd-exposed groups received Cd as CdCl_2_ (CdCl_2_, Sigma-Aldrich, St. Louis, MO, USA) in four different daily doses (0.09, 0.9, 1.8, and 4.5 mg Cd/kg b.w.), which corresponded to 1/1000, 1/100, 1/50, and 1/20 LD50 [65] (*n* = 8 each group) for 90 days. The doses of Cd were selected to represent environmental levels of exposure in the general population (0.09 and 0.9 mg Cd/kg b.w.) based on literature data [15]. The animals in the control groups received water by gavage; the volume was the same as the doses used in the Cd groups (Figure 5).

During and after treatment, all animals were observed carefully for mortality, body weight, and gross behavioral changes. All procedures conducted on rats were in accordance with the Local Animal Ethical Committee of the Medical University of Lodz (protocol number: LKE 14/LB481/DLZ/2012).

#### Euthanasia, Tissue Collection, and Preservation

Female rats were weighed and underwent euthanasia (heart puncture under light anesthesia) after 90 days of exposure, after 90 days post-exposure, and after 180 days post-exposure. Subsequently, whole blood was drawn into Vacutainer tubes for metal analysis (S-Monovette, Sarstedt), plasma was collected by whole blood centrifugation (3000× *g*/10 min, 4 °C), and the liver and kidneys were trimmed of fat and weighed. The tissues, whole blood, and plasma samples were stored in acid-washed tubes at −20 °C until Cd and trace elements (Zn, Cu, Ca, and Mg) analysis.

### 4.2. Biochemical Analysis

#### Determination of Cd, Zn, Cu, Ca, and Mg Concentrations

Elemental analyses were performed by flame atomic spectrometry (F-AAS) and flameless atomic spectrometry (GF-AAS). Instrumental conditions of F-AAS and GF-AAS were investigated for each inorganic element. A one-dimensional approach was used to optimize inter alia pyrolysis and atomization temperatures to achieve the level of accuracy required by European Communities Directive 96/23 [66]. Analyses were carried out using a standard solution for each analyte, consisting of ASTASOL-MIX (Analytical Ltd., Prague, Czech Republic) in 1% (*v*/*v*) HNO_3_ (J.T. Baker, Ultrex II Analyzed, Phillipsburg, NJ, USA). The tissue samples were digested with a microwave digestion system MARSXpress (CEM Corporation, Matthews, NC, USA) with 10% (*v*/*v*) nitric acid.

Analyses of Cd and Cu concentrations were prepared in duplicate in plasma, liver, and kidney tissues, using a Hitachi Z-8270 GFAAS (Hitachi, Ltd., Tokyo, Japan) with a Zeeman-type background correction, an autosampler, and a pyro-coated tube. 

In the liver and kidneys of each rat, the Cd bioaccumulation rate (percent of cumulative administration dose) (CR) was additionally calculated depending on the dose in the three study periods according to the following formula:

CR (%) = C − Ck/ Dc × 100(1)

C—metal concentration in the tissue of the test group;

Ck—metal concentration in the tissue of the control group;

Dc—cumulative dose of Cd after 90 days of rat exposure.

The levels of Zn, Ca, and Mg were also determined in the plasma, liver, and kidneys in duplicate using flame atomic absorption spectrometry on F-AAS (Avanta GM; GBC Scientific Equipment Pty Ltd., Melbourne, Australia). To assess Zn, Mg, and Ca, mineralized tissues were additionally diluted (two to eight times) in relation to the measured elements. For each sample, three parallel independent determinations were made, and the mean values were recorded. 

The concentrations of Cd and trace elements in the tissues and plasma were reported as μg/g wet tissue and mg/L, respectively. The analytical quality control was within the range of reference values; reference samples were Seronorm Serum-1 (Sero, Billingstad, Norway) and Bovine Liver (No. 1577b; National Institute of Standards and Technology, Gaithersburg, MD, USA). For 96% of the determinations, the repeatability error did not exceed 10%. The detection limits (LOD) were as follows: Cd (0.02 μg/L or g wet tissue), Cu (0.09 mg/L or μg/g wet tissue), Zn (0.01 mg/L, Ca (0.07 mg/L), and Mg (0.3 mg/L or wet tissue).

### 4.3. Statistical Analyses

Statistica software version 9.1 (Statsoft Polska, Kraków, Poland) was used for statistical analyses. The statistical significance of any differences was calculated using the Kruskal–Wallis analysis of variance followed by the Mann–Whitney *U* test. Relationships were considered statistically significant at *p* ≤ 0.05. All results are expressed as mean ± SD (standard deviation).

## 5. Conclusions

In summary, our results indicate that after six months of observation, the greatest Cd accumulation in the kidneys occurs following low environmental exposure; hence, exposure to low doses of Cd appears to be particularly dangerous to the kidneys. When higher doses were administered, about 40% lower Cd accumulation was noted in the kidneys compared to the lower doses, with the value being three times higher than in the liver. 

The accumulation of Cd in the liver and kidneys induced significant disturbances in essential element homeostasis in a dose- and tissue-concentration-dependent manner. Cd concentrations > 21 μg/g w.w. resulted in increased Cu concentrations in the kidney, while Cd concentrations > 6 μg/g w.w. increased Cu and Zn concentrations in the liver. Our findings reveal that these changes in element homeostasis persisted for up to six months after the termination of Cd exposure and that some of them were exacerbated: renal Cu concentration was found to increase over the observation period. Such long-term disturbances in Cu/Zn balance may increase oxidative stress and, thus, lipid peroxidation, which may be a potential early risk factor for renal dysfunction. Most importantly, our findings suggest that there is a need to review the acceptable level of environmental exposure to Cd and its critical concentration in the renal cortex, which are currently considered safe. 

## Figures and Tables

**Table 1:** Cd concentrations in plasma, liver, and kidney after 90 days of oral exposure to Cd and after the 90 or 180 days of observation.

	Cd Dose [mg/kg b.w.]	Cd in Plasma [μg/L]	Cd in Plasma/Cd in Whole Blood [%]	Cd in Liver [μg/g]	Cd in Kidneys [μg/g]
90 days of exposure	0	0.020 ± 0.003	10	0.02 ± 0.004	0.08 ± 0.02
	environmental doses				
	0.09	0.824 ± 0.236 ↑	78	0.61 ± 0.07 ↑	1.76 ± 0.22 ↑
	0.9	5.020 ± 0.795 ↑	54	6.60 ± 1.71 ↑	8.13 ± 0.49 ↑
	higher doses				
	1.8	9.736 ± 1.442 ↑	50	15.94 ± 1.61 ↑	21.42 ± 4.07 ↑
	4.5	20.948 ± 2.343 ↑	44	50.67 ± 9.43 ↑	39.54 ± 2.47 ↑
90 days of exposure + 90 days of observation	0	0.001 ± 0.001	0	0.05 ± 0.02	0.22 ± 0.03
	environmental doses				
	0.09	0.210 ± 0.013 ↑	51	0.41 ± 0.32 ↑	1.98 ± 0.26 ↑
	0.9	0.757 ± 0.260 ↑	57	6.22 ± 0.80 ↑	9.70 ± 2.71 ↑
	higher doses				
	1.8	1.228 ± 0.332 ↑	60	13.59 ± 5.68 ↑	34.62 ± 9.43 ↑
	4.5	2.352 ± 1.063 ↑	45	43.18 ± 12.19 ↑	44.60 ± 6.43 ↑
90 days of exposure + 180 days of observation	0	0.005 ± 0.001	4	0.03 ± 0.01	0.30 ± 0.08
	environmental doses				
	0.09	0.030 ± 0.022 ↑	12	0.71 ± 0.05 ↑	2.77 ± 1.24 ↑
	0.9	0.141 ± 0.050 ↑	17	6.65 ± 2.08 ↑	23.20 ± 2.38 ↑
	higher doses				
	1.8	1.036 ± 0.422 ↑	76	13.22 ± 2.20 ↑	38.57 ± 10.71 ↑
	4.5	2.216 ± 0.742 ↑	82	30.60 ± 2.02 ↑	92.66 ± 18.85 ↑

The Cd plasma: whole blood ratio was calculated based on the whole blood Cd values reported in Nasiadek et al. (2022) [31]. All values are presented as mean ± SD (*n* = 8); ↑ indicates a statistically significant (*p* ≤ 0.05) increase in comparison to the control group.

**Table 2:** Zn and Cu concentrations in plasma, liver, and kidney after 90 days of oral exposure to Cd and after 90 days or 180 days of observation.

	Cd Dose [mg/kg b.w.]	Zn in Plasma [mg/L]	Zn in Kidney [μg/g]	Zn in Liver [μg/g]	Cu in Plasma [mg/L]	Cu in Kidney [μg/g]	Cu in Liver [μg/g]	Cu/Zn Ratio in Plasma	Cu/Zn Ratio in Kidney	Cu/Zn Ratio in Liver
90 days of exposure	0	1.01 ± 0.11	41.54 ± 5.43	33.36 ± 2.65	1.63 ± 0.08	32.78 ± 5.05	5.40 ± 0.32	1.61	0.79	0.16
	environmental doses									
	0.09	0.95 ± 0.05	48.47 ± 5.19	34.32 ± 2.11	1.52 ± 0.17	38.82 ± 3.76	5.52 ± 0.33	1.60	0.80	0.16
	0.9	1.12 ± 0.11	48.73 ± 5.78	43.66 ± 4.42 ↑	1.79 ± 0.25	36.83 ± 4.05	6.13 ± 0.47 ↑	1.60	0.76	0.14
	higher doses									
	1.8	1.12 ± 0.19	51.61 ± 2.98 ↑	46.46 ± 2.84 ↑	1.79 ± 0.23	44.62 ± 2.30 ↑	6.21 ± 0.38 ↑	1.60	0.86	0.13
	4.5	1.20 ± 0.18	60.48 ± 4.60 ↑	56.06 ± 2.57 ↑	1.97 ± 0.12 ↑	61.49 ± 3.82 ↑	6.25 ± 0.36 ↑	1.64	1.02	0.11
90 days of exposure + 90 days of observation period	0	1.18 ± 0.12	39.64 ± 4.65	30.18 ± 3.86	1.60 ± 0.08	30.67 ± 4.05	5.46 ± 0.43	1.36	0.74	0.18
	environmental doses									
	0.09	1.03 ± 0.10	35.25 ± 1.52	28.83 ± 2.87	1.45 ± 0.11	29.24 ± 3.62	5.19 ± 0.45	1.41	0.88	0.18
	0.9	1.27 ± 0.11	36.82 ± 6.32	33.54 ± 1.44	1.62 ± 0.07	33.66 ± 5.62	5.70 ± 0.43	1.28	0.91	0.17
	higher doses									
	1.8	1.21 ± 0.09	41.98 ± 1.81	39.99 ± 7.16 ↑	1.67 ± 0.08	38.11 ± 4.26	5.64 ± 0.52	1.38	0.91	0.14
	4.5	1.15 ± 0.07	37.23 ± 1.79	52.35 ± 7.04 ↑	1.68 ± 0.11	43.41 ± 4.25 ↑	6.54 ± 0.47 ↑	1.46	1.17	0.12
90 days of exposure + 180 days of observation period	0	1.15 ± 0.07	35.62 ± 2.78	28.96 ± 3.37	1.92 ± 0.16	28.54 ± 3.43	5.23 ± 0.74	1.67	0.77	0.18
	environmental doses									
	0.09	1.16 ± 0.11	36.47 ± 4.85	27.73 ± 1.94	1.84 ± 0.16	26.92 ± 5.06	5.32 ± 0.55	1.59	0.74	0.19
	0.9	1.28 ± 0.20	40.76 ± 1.85	31.32 ± 3.69	2.08 ± 0.20 ↑	38.81 ± 5.25 ↑	6.00 ± 0.78	1.62	0.95	0.19
	higher doses									
	1.8	1.24 ± 0.12	44.26 ± 4.72	50.79 ± 4.07 ↑	1.98 ± 0.27	47.38 ± 4.99 ↑	7.22 ± 0.27 ↑	1.60	1.07	0.14
	4.5	1.08 ± 0.08	40.78 ± 3.43	54.70 ± 5.81 ↑	1.85 ± 0.08	51.87 ± 6.23 ↑	7.11 ± 0.58 ↑	1.71	1.27	0.13

All values are presented as mean ± SD (*n* = 8); ↑ indicates a statistically significant (*p* ≤ 0.05) increase in comparison to the control group.

**Table 3:** Ca and Mg concentrations in plasma, liver, and kidney after 90 days of oral exposure to Cd and after 90 days or 180 days of observation.

	Cd Dose [mg/kg b.w.]	Ca in Plasma [mg/L]	Ca in Kidney [μg/g]	Ca in Liver [μg/g]	Mg in Plasma [mg/L]	Mg in Kidney [μg/g]	Mg in Liver [μg/g]	Mg/Ca Ratio in Plasma	Mg/Ca Ratio in Kidney	Mg/Ca Ratio in Liver
90 days of exposure	0	69.22 ± 2.34	47.40 ± 4.28	12.42 ± 1.16	31.4 ± 2.43	410.02 ± 39.96	250.2 ± 15.56	0.45	8.65	20.14
	environmental doses									
	0.09	70.04 ± 4.04	45.46 ± 5.02	12.51 ± 0.68	30.36 ± 1.48	407.19 ± 35.73	257.19 ± 21.40	0.43	8.96	20.56
	0.9	74.55 ± 4.23	44.69 ± 2.22	12.32 ± 0.50	29.69 ± 2.99	379.28 ± 41.14	252.54 ± 14.56	0.40	8.49	20.50
	higher doses									
	1.8	78.99 ± 3.12 ↑	49.27 ± 4.67	11.33 ± 0.22	29.48 ± 1.33	385.37 ± 49.92	252.93 ± 14.83	0.37	7.82	22.32
	4.5	79.86 ± 3.46 ↑	43.50 ± 4.80	11.27 ± 0.30	30.33 ± 2.18	416.84 ± 36.79	243.26 ± 7.14	0.38	9.58	21.58
90 days of exposure + 90 days of observation	0	69.86 ± 1.64	46.48 ± 3.18	10.40 ± 1.70	27.97 ± 2.09	375.18 ± 33.07	277.95 ± 4.09	0.40	8.07	26.73
	environmental doses									
	0.09	67.49 ± 2.95	38.12 ± 3.59	9.59 ± 0.55	27.88 ± 2.00	318.47 ± 25.57	268.27 ± 15.43	0.41	8.35	27.97
	0.9	70.23 ± 4.28	37.06 ± 4.65	10.77 ± 0.89	26.36 ± 1.90	328.11 ± 31.39	277.81 ± 16.45	0.38	8.85	25.79
	higher doses									
	1.8	71.83 ± 3.80	44.03 ± 1.35	10.67 ± 1.61	26.26 ± 2.56	327.94 ± 25.30	294.23 ± 12.40	0.37	7.45	27.58
	4.5	77.53 ± 3.04 ↑	36.30 ± 2.72	10.01 ± 0.56	26.08 ± 2.63	321.84 ± 9.22	279.72 ± 19.38	0.34	8.87	27.94
90 days of exposure + 180 days of observation	0	69.75 ± 5.58	38.67 ± 4.71	10.33 ± 0.84	31.14 ± 4.54	375.88 ± 18.06	260.36 ± 17.31	0.45	9.72	25.2
	environmental doses									
	0.09	74.17 ± 5.09	34.38 ± 1.29	10.26 ± 0.74	30.42 ± 3.14	406.27 ± 7.92	276.17 ± 13.90	0.41	11.82	26.92
	0.9	73.41 ± 2.11	37.94 ± 4.37	10.02 ± 0.86	27.17 ± 1.78	418.64 ± 19.01	300.16 ± 40.41	0.37	13.11	29.96
	higher doses									
	1.8	73.84 ± 5.21	35.68 ± 5.14	12.00 ± 0.54 ↑	26.09 ± 1.61	418.00 ± 24.85	329.78 ± 26.50 ↑	0.35	11.72	27.48
	4.5	69.63 ± 3.04	33.30 ± 1.09	11.74 ± 0.91 ↑	28.14 ± 1.62	372.18 ± 27.04	324.99 ± 28.64 ↑	0.40	11.18	27.68

All values are presented as mean ± SD (*n* = 8); ↑ indicates a statistically significant (*p* ≤ 0.05) increase in comparison to the control group.

## Data Availability

Data are contained within this article and its Supplementary Materials, as well as available upon request.

## Supplementary Materials

The following supporting information can be downloaded at https://www.mdpi.com/article/10.3390/ijms25073829/s1.
