# Supplementary material for: Changes in Endogenous Essential Metal Homeostasis in the Liver and Kidneys during a Six-Month Follow-Up Period after Subchronic Cadmium Exposure
Source: Int J Mol Sci. 2024 Mar 29;25(7):3829. doi: 10.3390/ijms25073829 (PMC11011286; doi:10.3390/ijms25073829)
Supplement: Supplementary file 1 [file ijms-25-03829-s001.zip › ijms-2908508-supplementary.docx]

**CHANGES IN ENDOGENOUS ESSENTIAL METAL HOMEOSTASIS IN THE LIVER AND KIDNEYS DURING A SIX-MONTH FOLLOW-UP PERIOD AFTER SUBCHRONIC CADMIUM EXPOSURE**

**Rafał Kusak^1^, Marzenna Nasiadek*^2^, Joanna Stragierowicz^2^, Wojciech Hanke^3^, Anna Kilanowicz^2^**

**Supplementary materials**

^1^ Medical Centers the Medici, Bazarowa 9, 91-053 Lodz, Poland

^2^ Department of Toxicology, Medical University of Lodz, Muszynskiego 1, 90-151 Lodz, Poland;

^3^ Medical Informatics and Statistics Department, Medical University of Lodz, Al. Kosciuszki 4, 90-419 Lodz, Poland

e-mail: [marzenna.nasiadek@umed.lodz.pl](mailto:marzenna.nasiadek@umed.lodz.pl); joanna.stragierowicz@umed.lodz.pl; [wojciech.hanke@umed.lodz.pl](mailto:wojciech.hanke@umed.lodz.pl); [anna.kilanowicz@umed.lodz.pl](mailto:anna.kilanowicz@umed.lodz.pl)

**Corresponding author.** Address: Marzenna Nasiadek, Department of Toxicology,

Faculty of Pharmacy, Muszynskiego 1, 90-151 Lodz, Poland;

tel./fax: +48 42 677 91 48

e-mail address: [marzenna.nasiadek@umed.lodz.pl](mailto:marzenna.nasiadek@umed.lodz.pl)

**Table S1.** Water intake [ml/24h/rat] following 90-day oral exposure to Cd.

**Table S2.** Food intake [g/24h/rat] following 90-day oral exposure to Cd.

**Table S3**. Water intake [ml/24h/rat] following 90-day oral exposure to Cd, and also after a 90 days observation period.

**Table S4**. Food intake [g/24h/rat] following 90-day oral exposure to Cd, and also after a 90 days observation period.

**Table S5**. Water intake [ml/24h/rat] following 90-day oral exposure to Cd, and also after a 180-day observation period.

**Table S6**. Food intake [g/24h/rat] following 90-day oral exposure to Cd, and also after a 180-day observation period.

**Table S7.** Changes in body weight, liver and kidneys mass following 90-day oral exposure to Cd, and also after a 90 or 180-day observation period.

**Table S1.** Water intake [ml/24h/rat] following 90-day oral exposure to Cd.

|  | **Week** | **Cd dose [mg/kg b.w.]** | | | | |
| --- | --- | --- | --- | --- | --- | --- |
|  |  |  | **environmental doses** | | **higher doses** | |
|  |  | **0** | **0.09** | **0.9** | **1.8** | **4.5** |
| **90-day exposure period** | 1 | 35.6 | 35.0 | 32.5 | 43.3 | 32.2 |
|  | 2 | 42.2 | 47.5 | 47.5 | 51.1 | 46.7 |
|  | 3 | 35.6 | 37.5 | 38.8 | 37.8 | 33.3 |
|  | 4 | 36.7 | 40.0 | 43.8 | 37.8 | 36.7 |
|  | 5 | 36.7 | 36.3 | 32.5 | 34.4 | 31.1 |
|  | 6 | 33.3 | 31.3 | 32.5 | 34.4 | 31.1 |
|  | 7 | 33.3 | 37.5 | 35.0 | 33.3 | 32.2 |
|  | 8 | 35.6 | 35.0 | 37.5 | 34.4 | 31.1 |
|  | 9 | 34.4 | 38.8 | 33.8 | 33.3 | 33.3 |
|  | 10 | 41.1 | 41.3 | 33.8 | 38.9 | 33.3 |
|  | 11 | 33.3 | 38.8 | 31.3 | 33.3 | 28.9 |
|  | 12 | 38.9 | 38.8 | 40.0 | 50.0 | 38.9 |
|  | 13 | 35.9 | 39.9 | 34.7 | 38.9 | 35.2 |
| **Mean water intake [ml/24h/rat]** | | 36.4 ± 3.0 | 38.2 ± 4.0 | 36.6 ± 5.1 | 38.5 ± 6.4 | 34.1 ± 4.8 |

**Table S2.** Food intake [g/24h/rat] following 90-day oral exposure to Cd.

|  | **Week** | **Cd dose [mg/kg b.w.]** | | | | |
| --- | --- | --- | --- | --- | --- | --- |
|  |  |  | **environmental doses** | | **higher doses** | |
|  |  | **0** | **0.09** | **0.9** | **1.8** | **4.5** |
| **90-day exposure period** | 1 | 16.9 | 16.1 | 17.1 | 17.4 | 16.1 |
|  | 2 | 18.6 | 21.8 | 24.9 | 24.0 | 22.9 |
|  | 3 | 15.4 | 15.6 | 14.8 | 16.7 | 12.2 |
|  | 4 | 15.6 | 21.9 | 20.4 | 20.6 | 18.4 |
|  | 5 | 14.4 | 14.1 | 15.4 | 15.2 | 13.3 |
|  | 6 | 14.2 | 13.4 | 14.8 | 16.4 | 16.9 |
|  | 7 | 15.6 | 14.6 | 14.9 | 16.0 | 11.7 |
|  | 8 | 16.4 | 13.5 | 15.9 | 18.6 | 12.3 |
|  | 9 | 16.4 | 16.3 | 19.4 | 19.8 | 11.8 |
|  | 10 | 13.7 | 12.9 | 14.1 | 16.2 | 12.4 |
|  | 11 | 12.0 | 13.4 | 14.0 | 14.0 | 13.8 |
|  | 12 | 15.7 | 13.4 | 16.8 | 22.1 | 11.9 |
|  | 13 | 13.5 | 15.6 | 15.2 | 19.3 | 12.5 |
| **Mean food intake**  **[g/24h/rat]** | | 15.3 ± 1.7 | 15.6 ± 3.0 | 16.7 ± 3.1 | 18.2 ± 2.9 | 14.3 ± 3.4 |

**Table S3.** Water intake [ml/24h/rat] following 90-day oral exposure to Cd, and also after a 90-day observation period.

|  | **Week** | **Cd dose [mg/kg b.w.]** | | | | |
| --- | --- | --- | --- | --- | --- | --- |
|  |  |  | **environmental doses** | | **higher doses** | |
|  |  | **0** | **0.09** | **0.9** | **1.8** | **4.5** |
| **90-day exposure period** | 1 | 40.0 | 45.0 | 35.6 | 42.2 | 38.9 |
|  | 2 | 28.9 | 32.5 | 37.8 | 42.2 | 23.3 |
|  | 3 | 41.1 | 35.0 | 34.4 | 38.9 | 28.9 |
|  | 4 | 25.6 | 32.5 | 28.9 | 33.3 | 22.2 |
|  | 5 | 28.9 | 31.2 | 28.9 | 32.2 | 27.8 |
|  | 6 | 26.7 | 26.2 | 27.8 | 18.9 | 27.8 |
|  | 7 | 30.0 | 28.7 | 27.8 | 25.6 | 26.7 |
|  | 8 | 27.8 | 31.2 | 30.0 | 24.4 | 24.4 |
|  | 9 | 35.6 | 33.7 | 33.3 | 28.9 | 36.7 |
|  | 10 | 27.8 | 31.2 | 32.2 | 27.8 | 32.2 |
|  | 11 | 42.2 | 37.5 | 41.1 | 37.8 | 44.4 |
|  | 12 | 33.3 | 27.5 | 27.8 | 37.8 | 28.9 |
|  | 13 | 31.2 | 29.6 | 32.6 | 31.2 | 33.8 |
| **90-day observation** | 14 | 45.6 | 30.0 | 32.2 | 31.1 | 42.2 |
|  | 15 | 28.9 | 31.2 | 30.0 | 35.6 | 31.1 |
|  | 16 | 36.7 | 28.7 | 33.3 | 31.1 | 35.6 |
|  | 17 | 29.6 | 29.6 | 33.7 | 31.9 | 36.4 |
|  | 18 | 36.7 | 31.2 | 32.2 | 32.2 | 37.5 |
|  | 19 | 30.0 | 36.2 | 35.7 | 33.0 | 30.5 |
|  | 20 | 38.2 | 41.2 | 35.5 | 41.0 | 32.7 |
|  | 21 | 44.2 | 32.5 | 30.7 | 33.2 | 32.2 |
|  | 22 | 35.7 | 28.7 | 32.5 | 35.0 | 30.5 |
|  | 23 | 29.6 | 35.7 | 30.2 | 37.5 | 32.6 |
|  | 24 | 31.9 | 32.9 | 33.7 | 32.7 | 33.7 |
|  | 25 | 39.6 | 39.6 | 35.9 | 33.4 | 35.9 |
|  | 26 | 32.7 | 31.5 | 34.6 | 35.4 | 31.4 |
| **Mean water intake [ml/24h/rat]** | | 33.7 ± 5.8 | 32.7 ± 4.4 | 32.6 ± 3.3 | 33.2 ± 5.4 | 32.2 ± 5.4 |

**Table S4.** Food intake [g/24h/rat] following 90-day oral exposure to Cd, and also after a 90-day observation period.

|  | **Week** | **Cd dose [mg/kg b.w.]** | | | | |
| --- | --- | --- | --- | --- | --- | --- |
|  |  |  | **environmental doses** | | **higher doses** | |
|  |  | **0** | **0.09** | **0.9** | **1.8** | **4.5** |
| **90-day exposure period** | 1 | 20.4 | 25.6 | 17.6 | 19.4 | 12.8 |
|  | 2 | 16.2 | 13.7 | 14.8 | 15.6 | 18.9 |
|  | 3 | 19.2 | 17.4 | 17.4 | 19.4 | 13.4 |
|  | 4 | 14.9 | 15.7 | 15.2 | 15.6 | 12.2 |
|  | 5 | 14.7 | 13.9 | 14.4 | 17.1 | 14.8 |
|  | 6 | 15.6 | 16.5 | 12.6 | 12.3 | 11.3 |
|  | 7 | 11.1 | 18.4 | 14.6 | 16.4 | 17.1 |
|  | 8 | 12.0 | 18.4 | 17.4 | 15.0 | 15.8 |
|  | 9 | 12.3 | 14.4 | 13.7 | 15.6 | 15.8 |
|  | 10 | 19.6 | 13.7 | 12.1 | 13.8 | 12.7 |
|  | 11 | 12.4 | 17.6 | 18.8 | 18.9 | 19.2 |
|  | 12 | 12.4 | 13.7 | 12.0 | 13.3 | 14.3 |
|  | 13 | 13.6 | 15.2 | 14.3 | 14.9 | 16.5 |
| **90-day observation** | 14 | 17.7 | 18.6 | 17.4 | 16.8 | 16.8 |
|  | 15 | 16.7 | 17.2 | 15.3 | 16.2 | 14.0 |
|  | 16 | 15.6 | 15.9 | 14.1 | 16.9 | 16.3 |
|  | 17 | 14.6 | 15.6 | 16.7 | 17.9 | 18.2 |
|  | 18 | 15.4 | 14.2 | 17.8 | 18.6 | 17.6 |
|  | 19 | 15.7 | 16.1 | 15.3 | 14.8 | 15.1 |
|  | 20 | 15.8 | 11.5 | 10.0 | 15.7 | 15.2 |
|  | 21 | 16.8 | 15.4 | 14.0 | 18.6 | 14.7 |
|  | 22 | 16.3 | 15.6 | 14.9 | 17.0 | 16.5 |
|  | 23 | 14.5 | 13.6 | 14.2 | 16.3 | 15.3 |
|  | 24 | 16.4 | 14.6 | 15.4 | 15.9 | 14.2 |
|  | 25 | 15.4 | 15.9 | 14.8 | 16.7 | 15.9 |
|  | 26 | 15.8 | 14.4 | 14.9 | 17.9 | 15.8 |
| **Mean food intake**  **[g/24h/rat]** | | 15.4 ± 2.3 | 15.9 ± 2.6 | 15.0 ± 2.0 | 16.4 ± 1.8 | 15.0 ± 2.2 |

**Table S5.** Water intake [ml/24h/rat] following 90-day oral exposure to Cd, and also after a 180-day observation period.

|  | **Week** | **Cd dose [mg/kg b.w.]** | | | | |
| --- | --- | --- | --- | --- | --- | --- |
|  |  |  | **environmental doses** | | **higher doses** | |
|  |  | **0** | **0.09** | **0.9** | **1.8** | **4.5** |
| **90-day exposure period** | 1 | 26.7 | 41.2 | 36.2 | 31.2 | 27.8 |
|  | 2 | 45.6 | 37.5 | 42.5 | 55.0 | 33.3 |
|  | 3 | 33.3 | 40.0 | 45.0 | 40.0 | 31.1 |
|  | 4 | 37.8 | 45.0 | 37.5 | 41.4 | 33.3 |
|  | 5 | 33.3 | 35.0 | 32.5 | 38.6 | 30.0 |
|  | 6 | 22.2 | 31.2 | 33.7 | 31.4 | 31.1 |
|  | 7 | 28.9 | 27.5 | 27.5 | 30.0 | 33.3 |
|  | 8 | 30.0 | 30.0 | 32.5 | 38.6 | 32.2 |
|  | 9 | 35.6 | 31.2 | 32.5 | 34.3 | 32.2 |
|  | 10 | 38.9 | 35.0 | 33.7 | 37.1 | 34.4 |
|  | 11 | 31.1 | 28.7 | 31.2 | 31.4 | 33.3 |
|  | 12 | 35.6 | 35.0 | 32.5 | 31.4 | 36.7 |
|  | 13 | 33.9 | 34.5 | 31.2 | 29.5 | 34.0 |
| **180-day observation** | 14 | 28.9 | 31.2 | 30.0 | 28.6 | 28.9 |
|  | 15 | 32.6 | 31.6 | 32.6 | 35.9 | 29.5 |
|  | 16 | 40.0 | 31.2 | 33.7 | 38.6 | 30.0 |
|  | 17 | 33.3 | 33.7 | 25.0 | 28.6 | 31.1 |
|  | 18 | 33.3 | 40.0 | 26.2 | 28.6 | 33.3 |
|  | 19 | 33.5 | 41.2 | 27.5 | 42.5 | 37.2 |
|  | 20 | 33.7 | 40.0 | 36.2 | 39.9 | 35.0 |
|  | 21 | 36.7 | 43.7 | 33.7 | 43.3 | 37.2 |
|  | 22 | 35.5 | 32.5 | 32.5 | 37.9 | 28.2 |
|  | 23 | 34.8 | 30.6 | 33.6 | 34.8 | 28.6 |
|  | 24 | 33.2 | 28.7 | 33.7 | 32.1 | 29.2 |
|  | 25 | 34.8 | 35.2 | 32.6 | 34.1 | 30.9 |
|  | 26 | 35.7 | 38.7 | 31.2 | 36.7 | 31.8 |
|  | 27 | 45.2 | 41.2 | 31.2 | 41.7 | 42.0 |
|  | 28 | 42.0 | 37.5 | 27.5 | 35.4 | 32.2 |
|  | 29 | 21.7 | 33.7 | 20.0 | 25.8 | 27.5 |
|  | 30 | 32.5 | 36.2 | 26.5 | 24.2 | 36.2 |
|  | 31 | 31.2 | 39.0 | 27.9 | 29.5 | 36.1 |
|  | 32 | 31.2 | 43.7 | 30.0 | 36.7 | 35.7 |
|  | 33 | 28.2 | 33.7 | 30.0 | 35.4 | 28.7 |
|  | 34 | 29.2 | 35.0 | 28.7 | 33.3 | 27.2 |
|  | 35 | 28.4 | 33.0 | 29.7 | 32.6 | 22.4 |
|  | 36 | 29.2 | 32.5 | 28.7 | 32.1 | 20.7 |
|  | 37 | 28.5 | 31.2 | 31.5 | 33.6 | 33.2 |
|  | 38 | 29.6 | 32.9 | 30.8 | 31.2 | 29.6 |
|  | 39 | 30.5 | 33.6 | 31.0 | 30.8 | 31.7 |
| **Mean water intake [ml/24h/rat]** | | 33.0 ± 5.1 | 35.2 ± 4.5 | 31.6 ± 4.4 | 34.72 ± 5.71 | 31.7 ± 4.0 |

**Table S6.** Food intake [g/24h/rat] following 90-day oral exposure to Cd, and also after a 180-day observation period.

|  | **Week** | **Cd dose [mg/kg b.w.]** | | | | |
| --- | --- | --- | --- | --- | --- | --- |
|  |  |  | **environmental doses** | | **higher doses** | |
|  |  | **0** | **0.09** | **0.9** | **1.8** | **4.5** |
| **90-day exposure period** | 1 | 16.4 | 15.4 | 16.9 | 22.1 | 15.0 |
|  | 2 | 22.0 | 13.6 | 21.2 | 31.0 | 18.2 |
|  | 3 | 15.8 | 14.7 | 15.5 | 20.4 | 16.7 |
|  | 4 | 27.7 | 18.9 | 18.7 | 21.1 | 19.4 |
|  | 5 | 14.0 | 13.2 | 14.2 | 18.4 | 13.8 |
|  | 6 | 15.4 | 13.2 | 16.9 | 20.0 | 14.4 |
|  | 7 | 15.2 | 12.7 | 20.0 | 17.1 | 13.1 |
|  | 8 | 13.6 | 12.2 | 16.0 | 19.1 | 18.9 |
|  | 9 | 15.6 | 11.9 | 16.0 | 23.6 | 15.1 |
|  | 10 | 15.1 | 13.4 | 15.0 | 17.9 | 15.1 |
|  | 11 | 13.3 | 12.7 | 14.2 | 12.9 | 12.6 |
|  | 12 | 19.3 | 13.9 | 14.2 | 15.4 | 17.7 |
|  | 13 | 16.2 | 12.6 | 14.6 | 13.6 | 15.6 |
| **180-day observation** | 14 | 13.6 | 12.1 | 14.1 | 15.3 | 15.6 |
|  | 15 | 15.8 | 13.6 | 17.9 | 16.2 | 15.6 |
|  | 16 | 17.4 | 16.5 | 19.1 | 21.1 | 16.4 |
|  | 17 | 14.8 | 14.2 | 14.5 | 23.0 | 14.9 |
|  | 18 | 14.4 | 19.1 | 15.4 | 18.9 | 18.0 |
|  | 19 | 14.9 | 13.9 | 15.0 | 18.9 | 15.6 |
|  | 20 | 16.0 | 15.1 | 15.2 | 17.1 | 16.4 |
|  | 21 | 14.2 | 14.7 | 14.0 | 17.9 | 15.9 |
|  | 22 | 14.3 | 16.7 | 13.6 | 15.9 | 21.7 |
|  | 23 | 14.5 | 15.6 | 13.8 | 16.3 | 17.9 |
|  | 24 | 14.2 | 16.7 | 14.4 | 15.6 | 14.8 |
|  | 25 | 14.2 | 14.9 | 14.3 | 15.7 | 14.2 |
|  | 26 | 14.0 | 13.6 | 14.1 | 16.1 | 14.0 |
|  | 27 | 14.6 | 15.9 | 15.5 | 15.7 | 16.4 |
|  | 28 | 14.7 | 13.9 | 13.6 | 14.9 | 14.0 |
|  | 29 | 15.2 | 14.1 | 16.4 | 18.5 | 13.8 |
|  | 30 | 20.6 | 17.1 | 13.0 | 17.3 | 15.4 |
|  | 31 | 18.6 | 15.6 | 14.2 | 16.8 | 13.7 |
|  | 32 | 14.5 | 14.1 | 16.0 | 15.5 | 12.7 |
|  | 33 | 13.7 | 15.9 | 15.0 | 19.0 | 15.3 |
|  | 34 | 12.8 | 13.7 | 13.9 | 16.7 | 12.0 |
|  | 35 | 15.3 | 13.9 | 14.2 | 16.7 | 12.4 |
|  | 36 | 17.4 | 14.7 | 13.7 | 16.8 | 12.9 |
|  | 37 | 16.2 | 14.9 | 13.8 | 17.9 | 13.5 |
|  | 38 | 15.9 | 15.3 | 13.7 | 18.6 | 14.9 |
|  | 39 | 14.6 | 14.4 | 14.5 | 18.4 | 14.9 |
| **Mean food intake**  **[g/24h/rat]** | | 15.8 ± 2.7 | 14.6 ± 1.7 | 15.3 ± 1.9 | 18.05 ± 3.20 | 15.3 ± 2.1 |

**Table S7.** Changes in body weight, liver and kidneys mass following 90-day oral exposure to Cd, and also after a 90 or 180-day observation period. All values are presented as mean ± SD (n=8)

|  | Cd dose [mg/kg b.w.] | Body weight | | | | Liver weight [g] | Relative liver weight [%] | Kidneys weight [g] | Relative kidney weight [%] |  |
| --- | --- | --- | --- | --- | --- | --- | --- | --- | --- | --- |
|  |  | At the beginning [g] | | | At the end [g] |  |  |  |  |  |
| 90-day exposure | 0 | 215.3 ± 4.11 | | | 234.6 ± 4.28 | 6.83 ± 0.90 | 2.91 ± 0.34 | 1.20 ± 0.11 | 0.51 ± 0.05 |  |
|  |  | |  | environmental doses | | | | |  |  |
|  | 0.09 | 216.5 ± 15.22 | | | 228.4 ± 14.29 | 6.44 ± 0.51 | 2.82 ± 0.18 | 1.17 ± 0.13 | 0.51 ± 0.04 |  |
|  | 0.9 | 235.2 ± 6.95 | | | 249.6 ± 7.09 | 7.12 ± 0.56 | 2.85 ± 0.16 | 1.34 ± 0.14 | 0.54 ± 0.05 |  |
|  |  | |  | higher doses | | | | |  |  |
|  | 1.8 | 234.7 ± 12.52 | | | 247.8 ± 13.14 | 7.15 ± 0.41 | 2.89 ± 0.24 | 1.37 ± 0.09 | 0.56 ± 0.05 |  |
|  | 4.5 | 231.6 ± 12.96 | | | 245.8 ± 13.14 | 7.39 ± 0.55 | 3.03 ± 0.10 | 1.39 ± 0.08 | 0.57 ± 0.02 |  |
| 90-day exposure + 90-day observation period | 0 | 215.3 ± 17.64 | | | 256.2 ± 16.01 | 7.66 ± 3.58 | 2.93 ± 0.16 | 1.29 ± 0.05 | 0.50 ± 0.03 |  |
|  |  | |  | environmental doses | | | | |  |  |
|  | 0.09 | 212.1 ± 29.65 | | | 257.2 ± 28.09 | 8.15 ± 0.50 | 3.19 ± 0.31 | 1.39 ± 0.12 | 0.51 ± 0.04 |  |
|  | 0.9 | 226.5 ± 34.78 | | | 252.6 ± 36.44 | 7.28 ± 0.95 | 3.05 ± 0.18 | 1.31 ± 0.23 | 0.52 ± 0.04 |  |
|  |  | |  | higher doses | | | | |  |  |
|  | 1.8 | 223.7 ± 9.11 | | | 261.0 ± 8.68 | 7.98 ± 0.39 | 3.06 ± 0.10 | 1.42 ± 0.10 | 0.55 ± 0.04 |  |
|  | 4.5 | 236.7 ± 15.24 | | | 244.2 ± 16.61 | 7.62 ± 0.99 | 3.13 ± 0.43 | 1.33 ± 0.13 | 0.54 ± 0.03 |  |
| 90-day exposure + 180-day observation period | 0 | 213.3 ± 27.95 | | | 249.7 ± 28.88 | 7.47 ± 0.86 | 2.99 ± 0.11 | 1.25 0.17 | 0.49 ± 0.03 |  |
|  |  | |  | environmental doses | | | | |  |  |
|  | 0.09 | 227.4 ± 12.63 | | | 258.5 ± 10.66 | 7.15 ± 0.64 | 2.77 ± 0.30 | 1.27 ± 0.16 | 0.49 ± 0.05 |  |
|  | 0.9 | 248.3 ± 11.52 | | | 280.0 ± 10.66 | 8.01 ± 0.76 | 2.86 ± 0.10 | 1.37 0.06 | 0.52 ± 0.01 |  |
|  |  | |  | higher doses | | | | |  |  |
|  | 1.8 | 243.7 ± 29.63 | | | 277.4 ± 31.65 | 7.95 ± 0.66 | 2.88 ± 0.26 | 1.47 ± 0.19 | 0.53 ± 0.02 |  |
|  | 4.5 | 237.6 ± 8.45 | | | 265.2 ± 7.63 | 7.49 ± 0.65 | 2.82 ± 0.17 | 1.45 ± 0.07 | 0.55 ± 0.03 |  |
